# Using Bayes' Rule to Define the Value of Evidence from Syndromic Surveillance

**DOI:** 10.1371/journal.pone.0111335

**Published:** 2014-11-03

**Authors:** Mats Gunnar Andersson, Céline Faverjon, Flavie Vial, Loïc Legrand, Agnès Leblond

**Affiliations:** 1 Department of Chemistry, Environment and Feed Hygiene, The National Veterinary Institute, Uppsala, Sweden; 2 INRA UR346 Animal Epidemiology, VetagroSup, Marcy L'Etoile, France; 3 Veterinary Public Health Institute, DCR-VPH, Vetsuisse Fakultät, Bern, Switzerland; 4 LABÉO - Frank Duncombe, Unité Risques Microbiens (U2RM), EA 4655, Normandie Universite, Caen, Normandy, France; 5 Réseau d'EpidémioSurveillance en Pathologie Equine (RESPE), Caen, France; 6 INRA UR346 Animal Epidemiology et Département Hippique, VetAgroSup, Marcy L'Etoile, France; The Pirbright Institute, United Kingdom

## Abstract

In this work we propose the adoption of a statistical framework used in the evaluation of forensic evidence as a tool for evaluating and presenting circumstantial “evidence” of a disease outbreak from syndromic surveillance. The basic idea is to exploit the predicted distributions of reported cases to calculate the ratio of the likelihood of observing *n* cases given an ongoing outbreak over the likelihood of observing *n* cases given no outbreak. The likelihood ratio defines the Value of Evidence (V). Using Bayes' rule, the prior odds for an ongoing outbreak are multiplied by V to obtain the posterior odds. This approach was applied to time series on the number of horses showing clinical respiratory symptoms or neurological symptoms. The separation between prior beliefs about the probability of an outbreak and the strength of evidence from syndromic surveillance offers a transparent reasoning process suitable for supporting decision makers. The value of evidence can be translated into a verbal statement, as often done in forensics or used for the production of risk maps. Furthermore, a Bayesian approach offers seamless integration of data from syndromic surveillance with results from predictive modeling and with information from other sources such as disease introduction risk assessments.

## Introduction

Syndromic surveillance appeared in the late 1990's and is becoming more and more popular in a wide range of human public health issues such as seasonal disease surveillance [1] and digital disease surveillance [2]. The wider acceptance of the relevance of the “One Health” concept [3] amongst public health practitioners has led to an increased exchange of methodologies and disease control knowledge between the human medicine and the veterinary sides. In the last 5 years, researchers in veterinary medicine have been investigating the application of syndromic surveillance methods for the early detection of zoonotic and non-zoonotic diseases [4].

There is no unique definition of “syndromic surveillance” but it is commonly accepted that it focuses on data collected prior to clinical diagnosis or laboratory confirmation [5], [6]. It is therefore based on non-specific health indicators which result in a surveillance system with low specificity but allow the early detection of outbreaks without *a priori* considerations. This constitutes a major advantage over traditional approaches which focus on a disease, or a list of reportable diseases, and rely on the ability of clinicians to correctly diagnose cases, which may be difficult when faced with a rare or emerging disease [4]. Moreover, the systematic and continuous data collection and analysis processes reduce the impact of chronic under-reporting observed in classical passive surveillance systems and also increases the sensitivity of this method [4]. Syndromic surveillance does not replace traditional approaches to disease monitoring (e.g. risk-based, active etc…) but is seen as an interesting and complementary tool for outbreak detection with a low specificity but with better sensitivity and timeliness [7].

Current approaches used in syndromic surveillance first seek to define the normal properties of the syndrome time-series when no outbreak of disease is recorded [4], [6] in order to be able to detect abnormal events overlaid on top of the background noise during an outbreak situation. In traditional aberration detection methods, an alarm goes off when the observed data exceed the expected values from the population [4], [6]. Such algorithms have an epidemic threshold and provide a yes/no qualitative output: “No, there is no outbreak” or “Yes, something unusual is happening in the population”.

This black or white vision of the health of the population of concern is simple but it may not always be adequate or useful for decision makers who may often find themselves in grey areas (indicator values close to the epidemic threshold). Moreover, binary result can also be difficult to combine with other epidemiological knowledge such as a probability of disease introduction or other complex parameters which influence decision making [8]. The development of syndromic surveillance quantitative outputs, which are more objective, flexible and easily interpretable, is a promising area of research.

The art of presenting scientific evidence to decision makers has been more extensively studied in forensic sciences in which legal certainty requires statements that clearly specify how strong the evidence for/against an hypothesis is and how the expert reached that conclusion. In recent years, the state of the art in forensic interpretation has been to evaluate forensic evidence using likelihood ratios in the framework of Bayesian hypothesis testing. Within this framework, it evaluates the extent to which results from forensic investigations speak in favor of the prosecutors or defendants hypotheses [9], [10]. The Bayesian approach has been applied to a wide range of forensic problems including evidence based on DNA analysis [10], mass spectroscopy [11], transfer of glass, fibers and paint [10] and microbial counts [12]. However, although initially developed for the legal system, the approach has been identified as useful for supporting decision making in other situations such as the tracing of *Salmonella spp* [13].

The aim of this study is to test the applicability of the Bayesian likelihood ratio framework to the early detection of outbreaks in a syndromic surveillance system. Transferability of the method is demonstrated by using two examples based on real data coming from RESPE, the French surveillance network on equine diseases. The first example makes use of data on French horses presenting nervous symptoms (NeurSy) and aim to test the ability of our approach to detect simulated outbreaks of an exotic disease, West Nile Virus (WNV). West Nile disease is an important zoonotic disease and syndromic surveillance applied in horses could be used as an early warning system to protect the human population [14]. The second example focuses on data on French horses with respiratory symptoms (RespSy) and is used to detect outbreaks of divergent strains of equine influenza (New-Influenza), a non-zoonotic disease leading to vaccine failure [15]–[18].

## Materials and Methods

### Background theory and proposed framework

Forensic evaluation of evidence is based on Bayesian hypothesis testing. In a syndromic surveillance context, this would mean that, in a particular week, there are two mutually exclusive hypotheses that should be evaluated, for example: H_1_ “There is an ongoing outbreak of disease x” and H_0_ “There is NOT an ongoing outbreak of disease x”. Without any extra information, the relative probability of the two hypotheses may be expressed as the *a priori* odds:
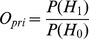
(Eq.1)where

P(H_1_): The *a priori* probability for hypothesis H_1_. Typically the probability of an ongoing outbreak of the disease of interest in a particular region.

P(H_0_): The *a priori* probability for hypothesis H_0_ which is the complementary hypothesis to H_1_. Typically the probability of an outbreak NOT going on.

In other words, the *a priori odds* define our *prior belief* about the disease status in the region. In a typical situation, the prior odds would be low (e.g. 1∶1000) but under some circumstances, it might be higher (e.g. if an outbreak is ongoing in a neighboring country). When we are presented evidence (E) of some kind pointing in favor (or against) of H_1_, this will make us update our belief. This posterior belief is expressed as the *a posteriori* odds. 
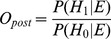
(Eq.2)


Where:

P(H_1_|E) is the probability of hypothesis H_1_, given the evidence (E).

P(H_0_|E) is the probability of hypothesis H_0_, given the evidence (E).

In syndromic surveillance, the evidence (E) is typically the number of reported suspected cases in a given time period. The degree to which the posterior belief differs from the prior belief will depend on the strength of the evidence. If the evidence is weak, the posterior odds will be similar to the prior odds whereas strong evidence in favor of H_1_ would result in posterior odds being much higher than the prior odds. At this point, it is important to note that the hypotheses to evaluate (H_1_) may differ and that the interpretation of the same piece of evidence would depend on the choice of H_1_. For example 10 reported cases of syndromes in horses may be a strong evidence that there is something unusual going on if these are nervous cases (H_1_  =  “ongoing outbreak of some nervous disease (i.e. WNV)”) but only weak evidence in favor of an equine influenza in the case of a respiratory syndrome (H_1_  =  “ongoing outbreak of equine influenza”), since in the latter case we might have expected far more reported cases.

This intuitive reasoning can be formalized by the application of Bayes' theorem:
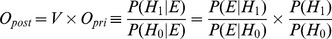
(Eq.3)


Where:

E is the number of reported cases of a syndrome in the particular week.

P(E|H_1_) is the probability of observing the evidence (E) given that H_1_ is true.

P(E|H_0_) is the probability of observing the evidence (E) given that H_0_ is true

In order to estimate P(E|H_1_) and P(E|H_0_) we need information on the probability distribution for the number of reported cases in a non-outbreak and outbreak situation. The probability of observing *n* cases given that H_1_ is true can be estimated using statistical modeling of baseline data [19]. When the cases are independent (i.e. not clustered), the data can be modeled using a general dynamic Poisson model [19]. When cases are clustered (overdispersion), the Poisson model will underestimate the probability of observing very high or very low number of cases, and in such cases, the data can be modeled by continuous mixtures of the Poisson distribution including Negative Binomial (NB) distribution or Poisson-log-normal (PLN) distribution [19].

The probability of E (observation of *n* cases) during an outbreak is calculated as:
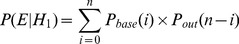
(Eq.4)


Where

P_base_(i)  =  Probability of drawing *i* cases from the baseline distribution (e.g. Poisson(λ) *or* NB(mu = mu_base_, size  =  theta_base_))

P_out_(i)  =  Probability of drawing *i* cases from the outbreak distribution (e.g. NB(mu = mu_out_, size  =  theta_out_))

The outbreak distribution may be estimated by fitting an appropriate probability distribution to data from historical outbreaks. In the absence of data, the outbreak distribution may be defined based on expert knowledge about the disease in question or assumptions about the distribution of a new disease. In most cases there would be a large uncertainty about the shape of the outbreak distribution.

**Figure 1:**
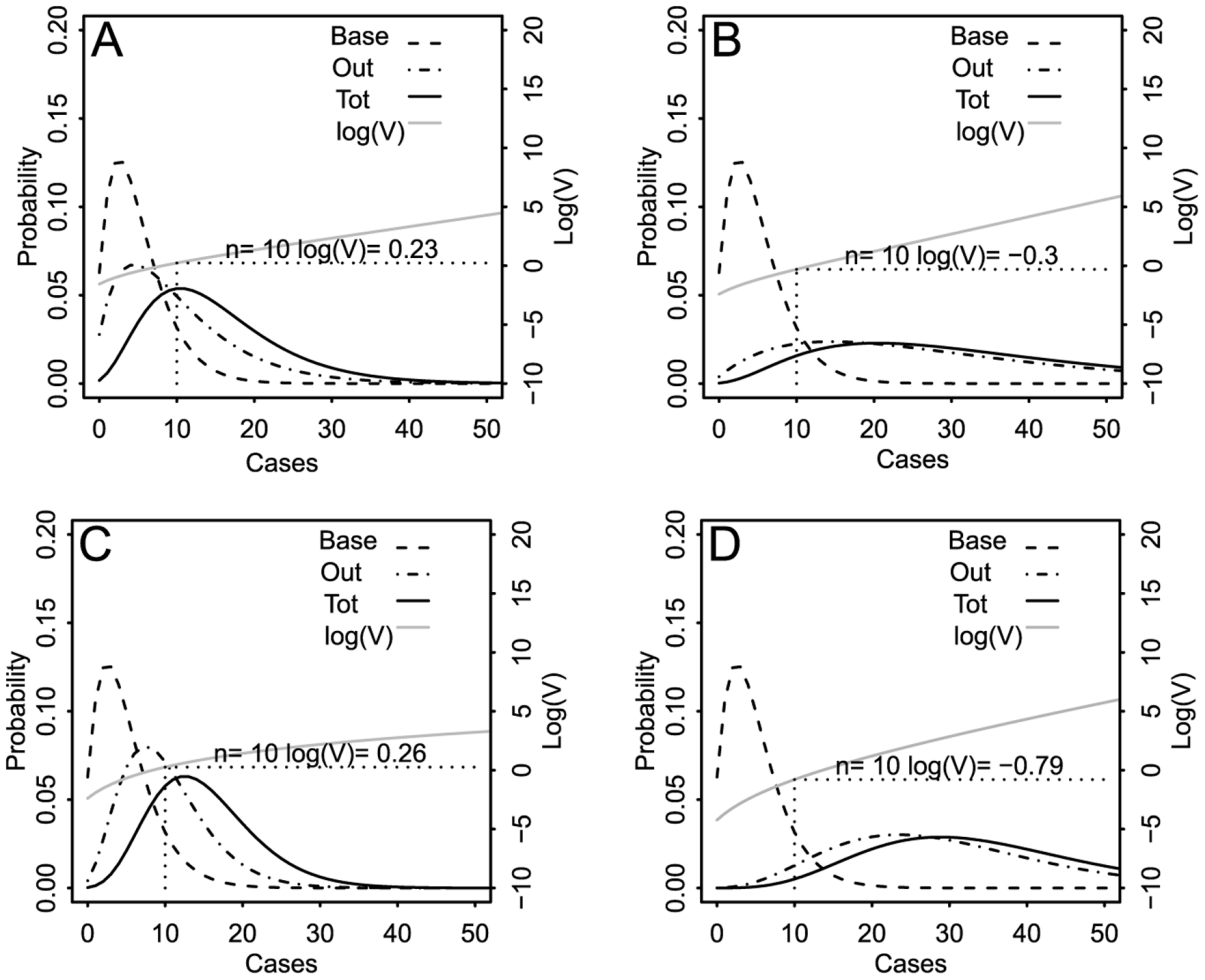
Value of evidence (V) and probability of observing 10 cases during a non-outbreak (Base) and outbreak situation (Out) with different assumptions about the magnitude of an outbreak. The baseline cases are distributed according to NB mu  = 5, theta  = 2.55. The value of evidence, log(V) is calculated as log_10_(p(n|outbreak)/p(n|baseline)). The distribution during an outbreak (Tot) is the sum of baseline cases and outbreak cases. In the examples A to D outbreak related cases are distributed according to (A) NB(mu = 10, theta  = 2), (B) NB(mu = 30, theta  = 2), (C) NB(mu = 10, theta  = 5), (D) NB(mu = 30, theta  = 5).

The next estimate is the probability of observing the Evidence (E) that is the actual number of reported cases. In forensics, the value of evidence (V) is defined as the ratio between the posterior and prior odds for H_1_ versus H_0_. The value of evidence (Fig. 1, line Log(V)) can be calculated from the two distributions by dividing the probabilities for each number of observed cases using equation 5:
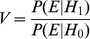
(Eq.5)


As illustrated in Fig 1 the value of evidence will depend on the assumptions about the outbreak. In the examples A to D, 10 cases are reported from a region where the baseline prevalence is around 5 cases per week. If it is expected that an outbreak may be small, resulting in only a small number of extra cases, 10 reported cases would speak in favor of an outbreak (Fig. 1, A, C). If, on the other hand, the disease(s) of interest are expected to yield a relatively large number of cases the evidence would speak against an outbreak (Fig. 1, B, D).

In addition, the strength of the evidence will depend on the precision on the estimates for the number of outbreak-related cases. If the distributions are wide (low theta, Fig 1A, 1B), the absolute value of log(V) is smaller whereas more narrow distributions (high theta, Fig 1C, 1D) result in higher values of log(V). This is intuitive: the more we know about what we expect to see during an outbreak, the stronger conclusions we will make from the observed evidence.

### Using the value of evidence for decision making

In contrast to traditional outbreak detection algorithms, the value of evidence approach does not have a built-in decision threshold. Typically a decision maker would not act upon syndromic surveillance data alone but rather combine it with other available knowledge. Cameron [20] proposed several approaches to disease freedom questions: (1) population or surveillance sensitivity, (2) probability of freedom from disease, and (3) expected cost of error – i.e., consequences of false positive and false negative results. All approaches underline how the value of inspection findings will be augmented when interpreted in a broader context to complement other monitoring and surveillance systems (MOSS) activities. One option for a decision maker would be to set an action threshold for the posterior odds. We might, for example, want to initiate an epidemiological investigation if the odds that there is an ongoing outbreak are larger than 1∶1 or 1∶100. Ideally the decision maker would make a cost-benefit analysis taking into account the expected costs for taking action versus not taking action. For example the decision maker may initiate control measures (vaccination program *etc*) when the odds are such that, on average, the reduced loss from the early detection of the outbreak would exceed the extra costs from initiating control measures (or vaccination programs) in response to false alarms.

The combination of evidence evaluation and decision theory is discussed in [21]. The expected utility (ū) of action *a_i_* is the average amount of loss that we expect to incur with this action. In the context of diseases surveillance, an action could be to implement movement restrictions, vaccination, sampling, control of vectors or to do nothing. The loss could be the direct financial losses (e.g. animal infection, disease and production losses) but also the indirect losses (e.g. surveillance and control costs, compensation costs, potential trade losses, social consequences). Since an unmanaged outbreak as well as actions will result in costs, the expected utility will always be zero or negative. In this framework the expected utility (ū) of action *a_i_* is defined as:
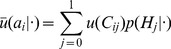
(Eq.6)where

H_1_  =  Outbreak

H_0_ =  No outbreak

*a_0_* =  No action

*a_1_* = Action

C_ij_ =  Different scenarios with respect to hypothesis on outbreak status (H_0_, H_1_) and action (a_0_, a_1_) C_00_ represents the case with no disease and no action implemented. C_01_ is no disease but action implemented, C_10_ disease but no action and C_11_ is disease and action implemented)

p(H_j_ |·) =  probability of hypothesis *j* given all available knowledge (Prior probability & evidence)

u(C_ij_)  =  expected utility for each possible situation C_ij_. Since gain is zero the utility is determined by economical and socio-economical loss.

According to this framework it is favorable to act when the expected utility of action (ū(*a_1_*|·)) is higher than the expected utility of no action (ū(*a_0_*|·). The relation between posterior probability (P(Hi|E) and posterior odds (O_post_) is defined by: 
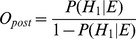
(Eq.7)and



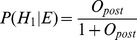
(Eq.8)Thus equation 6 can be reformulated as
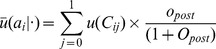
(Eq.9)


For each value of O_post_ the expected utility for action a_1_ and a_o_ is defined by eq. 9. The expected loss for each situation C_ij_ is based on expert opinion as indicated in table 1. An action threshold for posterior odds (O_post_*) can be defined as the value of O_post_ where 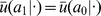



**Table 1:** Expected utility associated with different actions and the derived decision threshold & decision.

	Scenario A		Scenario B		Scenario C Large	
	Small outbreak in Autumn		Medium outbreak in Winter		outbreak in Spring	
u(C_00_) *Out*− *act*−	0		0		0	
u(C_10_) *Out*− *act+*	−0. 5 M€		−0. 5 M€		−0. 5 M€	
u(C_01_) *Out+ act*−	−5.1 M€		−5.3 M€		−10.1 M€	
u(C_11_) *Out+ act+*	−3.9 M€		−4.1 M€		−6.3 M€	
Action threshold Log_10_(O_post_*)	−0.38		−0.38		−0.88	
Log_10_(O_pri_)	−0.99		−3.03		−1.78	
Action Threshold Log_10_(V*)	0.61		2.65		0.9	
**Weeks**	**w36**	**w39**	**w1**	**w4**	**w25**	**w28**
Cases observed per week	3	4	5	7	5	7
Log_10_(V)	0.23	0.67	1.30	2.77	1.77	3.41
Log_10_(O_post_)	−0.76	−0.34	−1.71	−0.34	−0.01	1.63
Action? V>V*	**No**	**Yes**	**No**	**Yes**	**Yes**	**Yes**

In this work O_post_* was determined by numerical optimization. The derived action threshold for the value of evidence V* is calculated as:
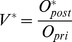
(Eq.10)where the prior odds for an ongoing outbreak Log_10_(O_pri_) is based on historical experience as well as knowledge about risk factors.

To make a decision, the risk manager would multiply the prior odds with the value of evidence using eq.3 to obtain the posterior odds for an outbreak O_post_(H_1_|E). If this odds goes over the action threshold Log_10_(O_post_*) where the expected utility from acting exceeds the utility for not acting, a decision would be taken to act.

### Performance assessment

Sensitivity, specificity and predictive values of positive and negative tests are important concepts when planning animal health monitoring. In the syndromic surveillance context a true positive (TP) is when the system alerts when an outbreaks is ongoing. A true negative (TN) is no alert and no outbreak. A false negative (FN) is when the system does not alert when an outbreak is ongoing, and, false positive (FP) is when the system alerts in the absence of an outbreak.

Sensitivity (SE) is the probability that a true outbreak triggers an alert:
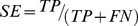
(Eq.11)


Specificity (SP) is the probability the there is no alert when no outbreak is ongoing:
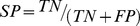
(Eq.12)


The positive predictive value (PPV) is the probability of an indicated outbreak being a true outbreak:
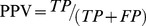
(Eq.13)


The Negative predictive value (NPV) is the probability that no signal of outbreak is true absence of an outbreak: 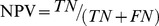
(Eq.14)


The PPV and NPV depend on the (prior) probability of an outbreak and in the performance assessment PPV was calculated as:
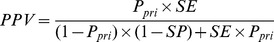
(Eq.15)where:

P_pri_ =  prior probability of ongoing outbreak in the week of interest

### Implementation

Models were implemented in R×64 version 3.0.2 [22]. TheR-Scripts are included as part of the material (Script S1, S2, S3, S4).

Dynamic regression was performed with function *glm* (package {stats) [22] for Poisson regression and *glm.nb* (package {MASS}) [23]. The expected number of counts at time × were estimated with the *predict* function of the respective package. Alternative regression models were evaluated using the Akaike information criterion (AIC). In addition adjusted deviance (Deviance/df) was used as a measure of goodness of fit (GOF).

The receiver operating characteristic (ROC) curve was generated in R by simulation. Counts for negative weeks were sampled from a Poisson distribution (function *rpois* in package {stats}) with lambda equal to the predicted value for each week in 2011 and 2012 (n = 53000). Counts for positive weeks were generated by sampling values from the fitted outbreak distribution (function *rnbinom* in package {stats}) and adding to the baseline.

SE and SP were calculated for values of Log10(V) between -1 and +3 in steps of 0.01. The expected PPV for each value of V was calculated as above using the prior odds for outbreak from three scenarios.

Threshold values for posterior odds (O_post_*) were estimated using the Solver function of Microsoft Excel 2007.

### Sources of data

As a proof of principle the value of evidence framework was applied to neurological and respiratory syndromes in French horses. The associated time series are named NeurSy and RespSy, respectively. These data are collected through the passive surveillance system “RESPE”, the French network for the surveillance of equine diseases (http://www.respe.net/). This system collects the declarations from veterinary practitioners registered as sentinels who fill online a standardized questionnaire depending on the syndrome concerned. Along with their declaration, veterinarians send standardized samples for the laboratory diagnosis. Tests for equine influenza, equine herpes 1 and 4 and equine arteritis viruses are implemented in the case of a respiratory syndrome, West Nile and equine herpes 1 viruses in the case of a nervous syndrome. In our study, we used these weekly time series.

Data from 2006 to 2010 were used to train our models and define the background noise of each time series when no outbreak occurs. We only used the data on the number of cases with no positive laboratory test result in order to remove the outbreaks from our datasets and obtain these outbreak free baselines. Then, different regression models were tested.

No real outbreak of West Nile disease and divergent strains of equine influenza (New-Influenza) occurred during this time. Instead fictive test data were used for demonstrating outbreak detection. The baselines in the test data were based on NeurSy and RespSy data from 2011 to 2012 where unexplained aberrations, not related to the diseases of interest, were filtered out and fictive outbreaks inserted based on historical data. The weekly counts from several real outbreaks were fitted together to model the outbreaks of each disease. The *prior odds* for each example are based on our knowledge on the epidemiology and risk factors for transmission of the disease. New-Influenza is supposed to have the same probability of occurrence over the year and the *prior odds* is thus considered as constant over time. West Nile disease transmission is linked to the vector's level of activity and is thus a seasonal disease. Different *prior odds* are set for each season for this disease.

### Data Accessibility

The datasets supporting this article have been uploaded as part of the Material. The baseline data for NeurSy and RespSy are included in Table S1 and Table S2 respectively. The outbreak data for NeurSy and RespSy are included in Table S3 and Table S4 respectively.

The software R can be freely downloaded from the CRAN homepage (http://cran.r-project.org/).

## Results

### Case study – Neurological syndromes and WNV (NeurSy)

#### Non-outbreak situation

To define the background noise of the NeurSy time series when no outbreak occurred, we fitted alternative regression models based on Poisson and NB distributions from years 2006–2010 on data containing only cases with no positive laboratory results (figure S1). The models evaluated including sinod models with 1, 2 and 3 periods/year and season or month as factorial variables. To account for differences between years we dynamically calculate the average counts for 53 consecutive weeks (*histmean*). To ensure that an ongoing outbreak will not influence the estimate, we used a 10 week guard band [24] for calculation of *histmean*. For the Poisson as well as the NB regression the best fit were obtained with the simplest model: 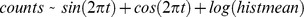
where t is time in years. For the Poisson regression we obtained: AIC = 637.8, GOF(adjusted dev)  = 1.156. For NB regression the corresponding parameters were: AIC: 639; GOF = 1.080. The inverse theta of the NB model was 10.45. Considering that the NB distribution converges to the Poisson distribution when inverse theta approaches infinity and that the GOF and AIC for the Poisson and NB models were very similar we conclude that the Poisson model adequately describes the random distribution in this data.

#### Outbreak definition

Three observed WNV outbreaks were used to simulated the outbreaks in our model: French outbreaks in horses in 2000 [25] and 2004 [14] where 76 and 32 confirmed cases were reported respectively among 131 and 72 horses presenting nervous symptoms, and the Italian outbreak in 1998 [26] where 14 cases of WNV in horses were investigated by week of onset.

The weekly counts from these three outbreaks were fitted to the NB distribution. The resulting outbreak distribution was NB(mu = 4.45, theta = 0.94). Based on this we predicted a median number of outbreak-related cases per week during an outbreak to be 3 with a 95% confidence interval of 0 to 18 cases.

#### Outbreak detection

**Figure 2:**
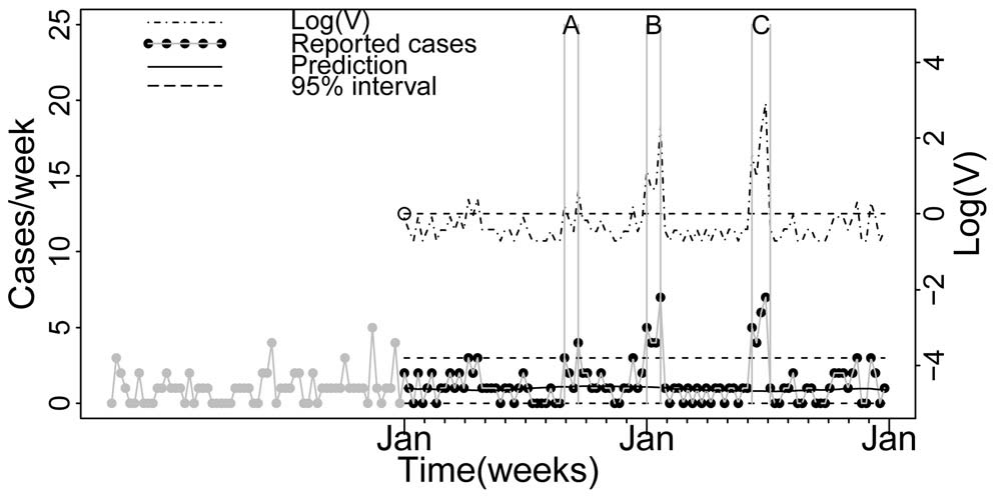
Application of NeurSy model on the test dataset. The vertical lines bounds peaks inserted during Year 1, week 36 to 39 (Scenario A), Year 2, week 1 to 4 (Scenario B) and Year 2, weeks 24 to 28 (Scenario C).

**Figure 3:**
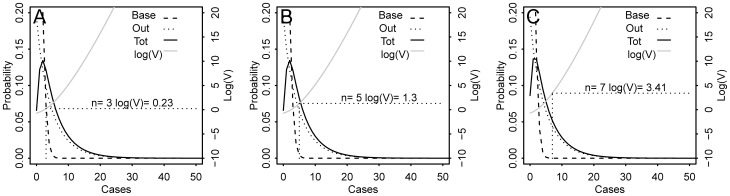
Value of evidence (V) and probability of observing *n* cases of neurological syndromes in a week during a non-outbreak (Base) situation and during a WNV outbreak (Out). Out is the distribution of outbreak related cases and Tot is the total number of observed cases per week during an outbreak. (A) Scenario A, year 1 week 36, λ = 1.08, (B) Scenario B, year 2 week 1. λ = 1.08, (C) Scenario C, year 2 week 27, λ = 0.81.

Three scenarios were tested. The probability of an outbreak is not constant over the year, instead the relative probability of an outbreak occurring in spring (week 10 to 30), summer/autumn (week 31 to 46) and winter (week 47 to 9) is approximately 1∶5∶0.04. We chose to test one scenario per time period. i.e. the scenario A occurs in autumn, scenario B in winter and the scenario C in spring. For each scenario, the Poisson model was applied on the test set and one simulated peak/outbreak was inserted into the baseline (Figure 2). For each week the value of evidence was calculated using Eq5 where the probability of the observed number of cases during no outbreak p(E|H_1_) and during outbreak p(E|H_0_) were calculated using the fitted model. Examples of the calculation of V during a non outbreak (scenario A) and during outbreaks (scenarios B and C) are shown in Figure 3.

### Decision scenarios

The decision making in the outbreak scenarios for both examples is summarized in table 1.

The expected utility u(C_ij_) for each scenario considered are given together with the action thresholds for posterior odds (O_post_*) and value of evidence (V*) in favor of an outbreak. That is the situation for which the decision to act and not act have the same expected utility.

The expected utility of taking action in response to false alert (u(C_01_)) represents the costs for increased surveillance and preventive actions such as mosquito control for WNV. The utility of not taking action when there is an outbreak (u(C_10_)) represents the costs for control and economical and socio-economical consequences of an outbreak when the response to the outbreak was delayed. The losses may depend on season and in the example we have assumed that a WNV outbreak in summer or spring in the south of France results in extra costs due to its impact on tourism. Finally the utility of taking action when there is an outbreak (u(C_11_)) represents the costs for surveillance plus the economical and socio-economical impact in case of a timely response to the outbreak.

For NeurSy (Scenarios A to C), the prior odds in the table are based on the assumption that an outbreak of WNV is likely to occur every 3 years over an averageof 5 weeks. The costs used are fictional but proportional to their expected relative contributions.

During the most at risk season regarding the probability of disease occurrence (Highest O_pri_), the alarm threshold is low and 4 cases are sufficient to trigger an action (See Table 1. scenario A). For the season less at resk, the expected utilities are similar than during the most at risk season (O_post_* are equal), but no action is implemented even if 7 cases are reported because they are unlikely due to WNV (Low O_pri_) (See Table 1. scenario B).

### Sensitivity, specificity and receiver operating characteristics

**Figure 4:**
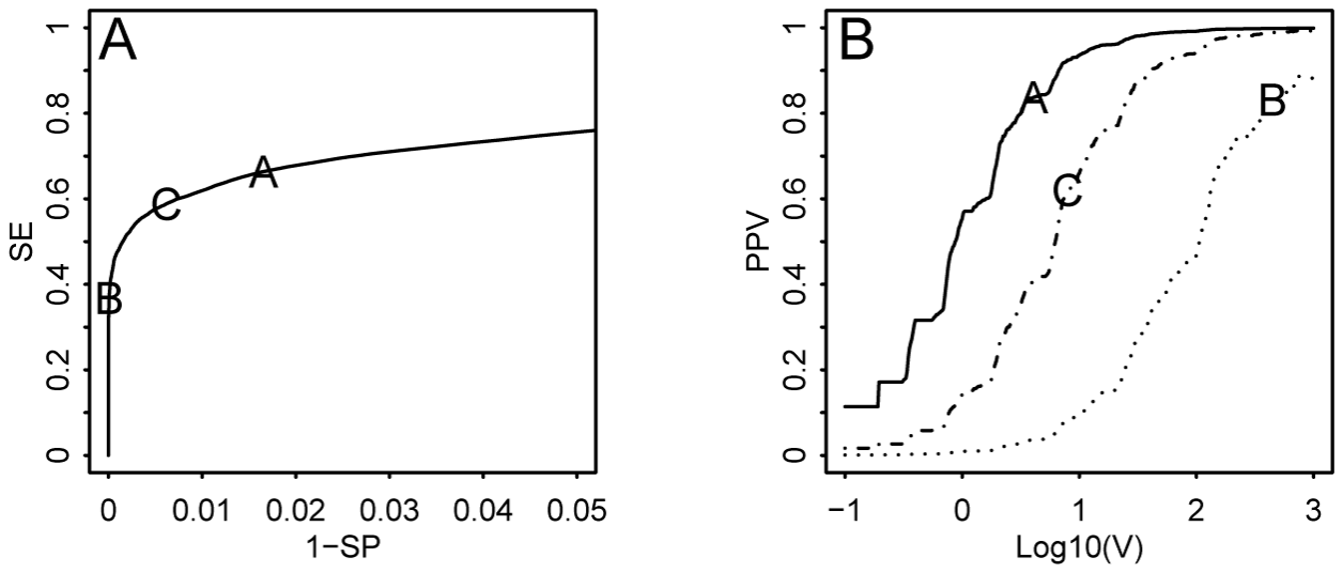
(A) ROC curve for outbreak detection of WNV based on neurological symptoms. Letters A–C indicate the decision threshold for Log(V*) in scenario A–C respectively (B). Positive Predictive Value (PPV) for different thresholds of Log_10_(V*) given the prior probabilities of scenario A, B and C. The position of the letters indicate the action threshold for the respective scenario.

The sensitivity and specificity of a surveillance system is defined by the chosen action threshold. The tradeoff between sensitivity and specificity of a model may be summarized in a receiver operating characteristics (ROC) curve [27]. The ROC curve corresponding to the case WNV case study is shown in figure 4A. The values of SE and SP arising from scenarios A to C are indicated by letters. The PPV i.e. the probability that an alarm corresponds to a real outbreak [28] depends not only on SE and SP but also on the prior probability of an outbreak as indicated in figure 4B.

### Case study 2– Respiratory syndromes and equine influenza (RespSy)

The same approach was successfully applied to the RespSy dataset. However, in this case the analysis indicated a significant degree of overdispersion in the weekly counts. Using the same regression model (counts ∼ sin(2π t) + cos(2πt) + log(histmean)) the NB model had lower AIC (1141 vs 1284) and GOF closer to one (1.14 vs 2.54) compared to the Poisson model. The theta parameter for the NB distribution was 1.78, and resulting in a much wider confidence interval for the expected number of cases in a non-outbreak situation (Figure S2) compared to the Poisson model (Figure S3). When the NB and Poisson models are applied to the same test dataset (Figure S4, S5) the latter will report a value of evidence for the inserted peaks (D, E) that is several orders of magnitude higher than does the NB model. The Poisson model also reports peaks with Log(V) close to 2 several times per year (Figure S5). An underlying assumption in the Poisson model is the absence of overdispersion and, when this assumption does not hold, the Poisson model underestimates the probability of obtaining a large number of reported cases in the non-outbreak situation. Consequently it overestimates the value of evidence in favor of an outbreak. The overdispersion may be due to clustering in reporting. In the surveillance protocol veterinarians are encouraged not only to declare the diseased horse but also 1 to 3 additional horses (from the same stable), suspected to be in the incubation phase of influenza.

## Discussion

In this work we have demonstrated how the value of evidence concept may be incorporated in a decision support system for syndromic surveillance and how the output may be used for risk assessment and informed decision making. According to the OIE - Terrestrial Animal Health Code [29] the decision to take action involves balancing costs for activities against economical and social consequences of a delayed response to an outbreak is the responsibility of the risk manager and should be separate from risk assessment.

Thus, although it is perfectly possible to build a system that outputs a best decision, the proposed approach is in concordance with the risk analysis framework [29] by offering explicit separation of assumptions (P_prior_), scientific evidence (V) and criteria for decisions and a transparency of how the evidence is evaluated. In forensics, the value of evidence is typically presented to the court as a qualitative statement in which fixed verbal expressions correspond to specified intervals for V [10], [30]. This approach may be useful also when presenting epidemiological results. For example a value of Log_10_(V) in the range 1–2 may be expressed as “results provide moderate evidence to support that an outbreak is ongoing”. Alternatively intervals for V and/or O_post_ could be expressed using a color scale to produce maps representing the results from surveillance and risk of ongoing outbreaks of different diseases.

The model presented here is intended as a proof of concept and when setting up an operational syndromic surveillance system it will, as usual, be necessary to perform a careful evaluation of the baseline model to ensure that the regression model does not overfit to the baseline data. When designing the current model it was evident that high dimensional regression models were prone to find artefactual seasonal patterns that could severely bias the estimated probability of observing a number of counts in a particular week (results not shown). In the current implementation the model learns seasonal patterns and distribution of residuals (Inverse theta parameter of NB distribution) from manually curated data whereas the expected yearly average (*histmean*) is continuously updated from outbreak-filtered weekly data. Naturally the value of evidence concept may also be applied to a system where the baseline model is automatically retrained on new data. However, since the distribution parameter (theta) of the NB distribution would determine the cutoff in the filtering algorithm we argue that it is safer not to use the filtered data for estimation of the same parameter without prior inspection of the data. The same conclusion holds for seasonal patterns.

The overdispersion in the RespSy dataset is largely due to veterinarians sampling several horses in a stable upon suspicion. Thus, in this special case it might be possible to handle the overdispersion by pre-processing the data to remove redundant cases, provided that the same pre-processing is applied to new data on weekly basis. However, when the mechanism behind overdispersion in baseline counts is not so transparent that automatic filtering out redundant cases is possible the NB model will support a correct interpretation of the value of the peak in the count data.

As indicated in Figure 4 the tradeoff between SE and SP differs between seasons. This is natural since in case the (prior) probability of an outbreak differs between seasons the average sensitivity SE_avr_ and specificity SP_avr_ will be given by:
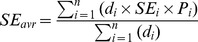
(Eq.16)

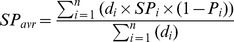
(Eq.17)where:

SE_i_ =  sensitivity in season i

SP_i_ =  specificity in season i

P_i_ =  (prior) probability of outbreak in season i

d_i_ =  (relative) duration of season i

Thus, by incorporating prior knowledge about the seasonality of the diseases of interest it is possible to achieve a high average sensitivity without sacrificing the PPV and SP. Another important attribute of outbreak detection is timeliness. Whereas there is no general measure of timeliness [28] the number of cases are often small in the first week(s) of an outbreak, increasing the sensitivity (i.e. lowering the threshold for V and thus *n*) in the high risk season will result in improved timeliness as well as average sensitivity.

In this work we have introduced the framework using models that evaluate evidence from each week independently. Although this simple approach is suitable for presenting the framework and a reasonable choice for an early warning system, the evaluation of evidence from one week at a time is not a fundamental limitation of the approach. A model accounting for accumulation of evidence over several weeks may, for example, be constructed by considering, for each week in the interval [0…j] the conditional probability
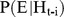



Where

t is the week of interest

H_t-i_ is the hypothesis that an outbreak started i weeks before t

E_t-n_ is the number of reported cases in week [t-i… t]

The probability of observing *n* outbreak-related cases will not be uniform throughout the outbreak but depend on whether the outbreak is in its first, second or third week *etc*. When accounting for evidence from several weeks the value of evidence in favor of the hypothesis H_1_ “An outbreak is going on” against H_0_ “An outbreak is not going on” will be dependent on the prior probability of an outbreak starting in any of the preceding weeks. This is due to the fact that H_1_ is composed of several sub-hypotheses:

H_1 i = o_: An outbreak started in week t

H_1 i = 1_: An outbreak started in week t-1

..

H_1 i = j_: An outbreak started in week t-j

Consequently p(H_1_) depends on the relative probability of these sub-hypotheses. The value of evidence in favor of an outbreak going on in week of interest (V) can be calculated as the Bayes factor (B):
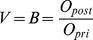
(Eq.18)where

O_post_ is the posterior odds of an outbreak going on in week of interest

O_pri_ is the prior odds of an outbreak going on in week of interest

Although in the more complex models the calculation of the value of evidence would depend on the prior probability of outbreak, the framework is still applicable for communicating the evidence to decision makers. Essentially any Markov Chain model could be applied in the evaluation of evidence framework and the choice of complexity is a tradeoff between on the one hand realism and on the other hand simplicity and transparency. However, we anticipate that in most situations there will not be sufficient data to support very complex models.

## Supporting Information

Figure S1 **Fitted baseline and one sided 95% confidence interval for weekly counts for case NeurSy Years 2006–2010.** Poisson regression using model: counts ∼ sin(2π t) + cos(2π t) + log(histmean).
(TIF)

Figure S2 **Fitted baseline and one sided 95% confidence interval for weekly counts for case RespSy Years 2006–2010. NB regression using model: counts ∼ sin(2π t) + cos(2π t) + log(histmean).**
(TIF)

Figure S3 **Fitted baseline and one sided 95% confidence interval for weekly counts for case RespSy Years 2006–2010.** Poisson regression using model: counts ∼ sin(2π t) + cos(2π t) + log(histmean).
(TIF)

Figure S4 **Application of RespSy NB-model on the fictive test dataset.** The vertical lines bounds peaks inserted during Year 1, week 36 to 39 (D), Year 2, week 24 to 28 (E). The gray points indicate historical data used to calculate the historical average (histmean).
(TIF)

Figure S5 **Application of RespSy Poisson-model on the fictive test dataset.** The vertical lines bounds peaks inserted during Year 1, week 36 to 39 (D), Year 2, week 24 to 28 (E). The gray points indicate historical data used to calculate the historical average (histmean).
(TIF)

Table S1 **NeurSy baseline 2006–2012.**
(CSV)

Table S2 **RespSy baseline 2006–2012.**
(CSV)

Table S3 **NeurSy outbreak distribution.**
(CSV)

Table S4 **RespSy outbreak distribution.**
(CSV)

Script S1 **Script used to analyze time series data.**
(R) Click here for additional data file.

Script S2 **Script used to illustrate the calculation of V in** **figures 1** **and** **3** **.**
(R) Click here for additional data file.

Script S3 **Script used to prepare ROC and PPV plots.**
(R) Click here for additional data file.

Script S4 **Script used to remove aberrations from baseline when preparing fictive test data.**
(R) Click here for additional data file.
