# Supplementary figures and images for: Using Bayes' Rule to Define the Value of Evidence from Syndromic Surveillance
Source: PLoS One. 2014 Nov 3;9(11):e111335. doi: 10.1371/journal.pone.0111335 (PMC4218722; doi:10.1371/journal.pone.0111335)

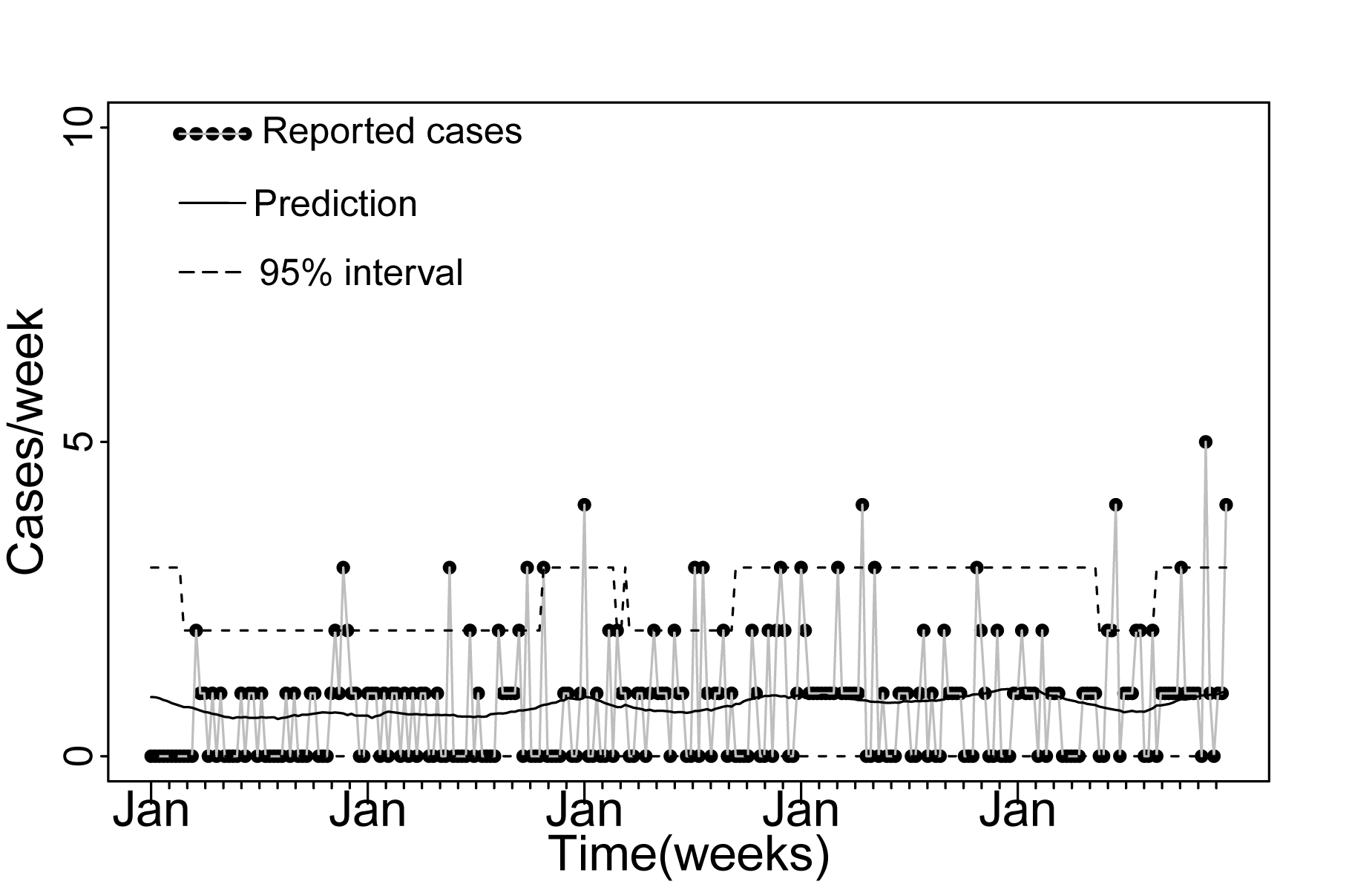

Supplement: Figure S1 — Fitted baseline and one sided 95% confidence interval for weekly counts for case NeurSy Years 2006–2010. Poisson regression using model: counts ∼ sin(2π t) + cos(2π t) + log(histmean). (TIF) [file pone.0111335.s001.tif]

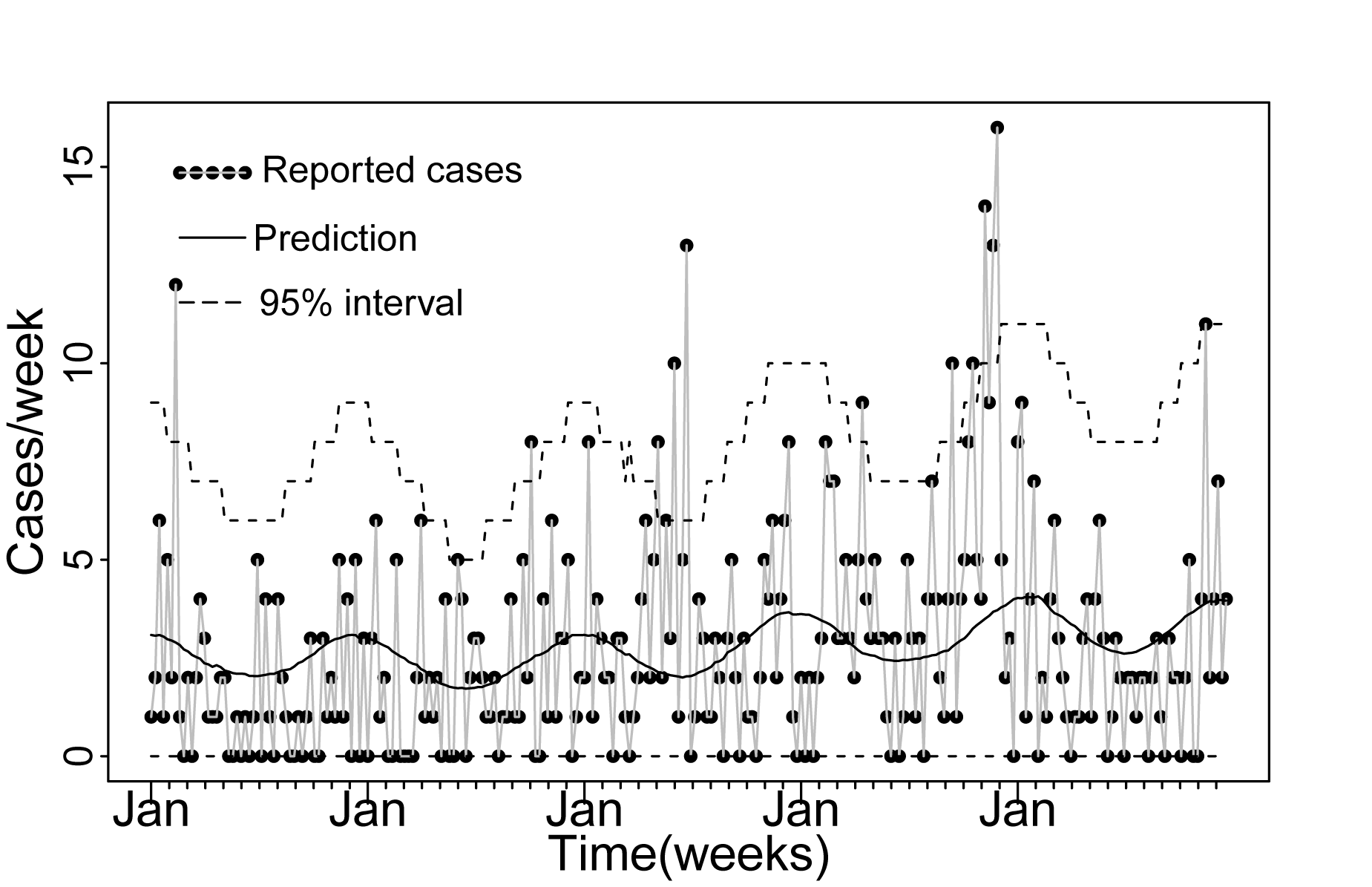

Supplement: Figure S2 — Fitted baseline and one sided 95% confidence interval for weekly counts for case RespSy Years 2006–2010. NB regression using model: counts ∼ sin(2π t) + cos(2π t) + log(histmean). (TIF) [file pone.0111335.s002.tif]

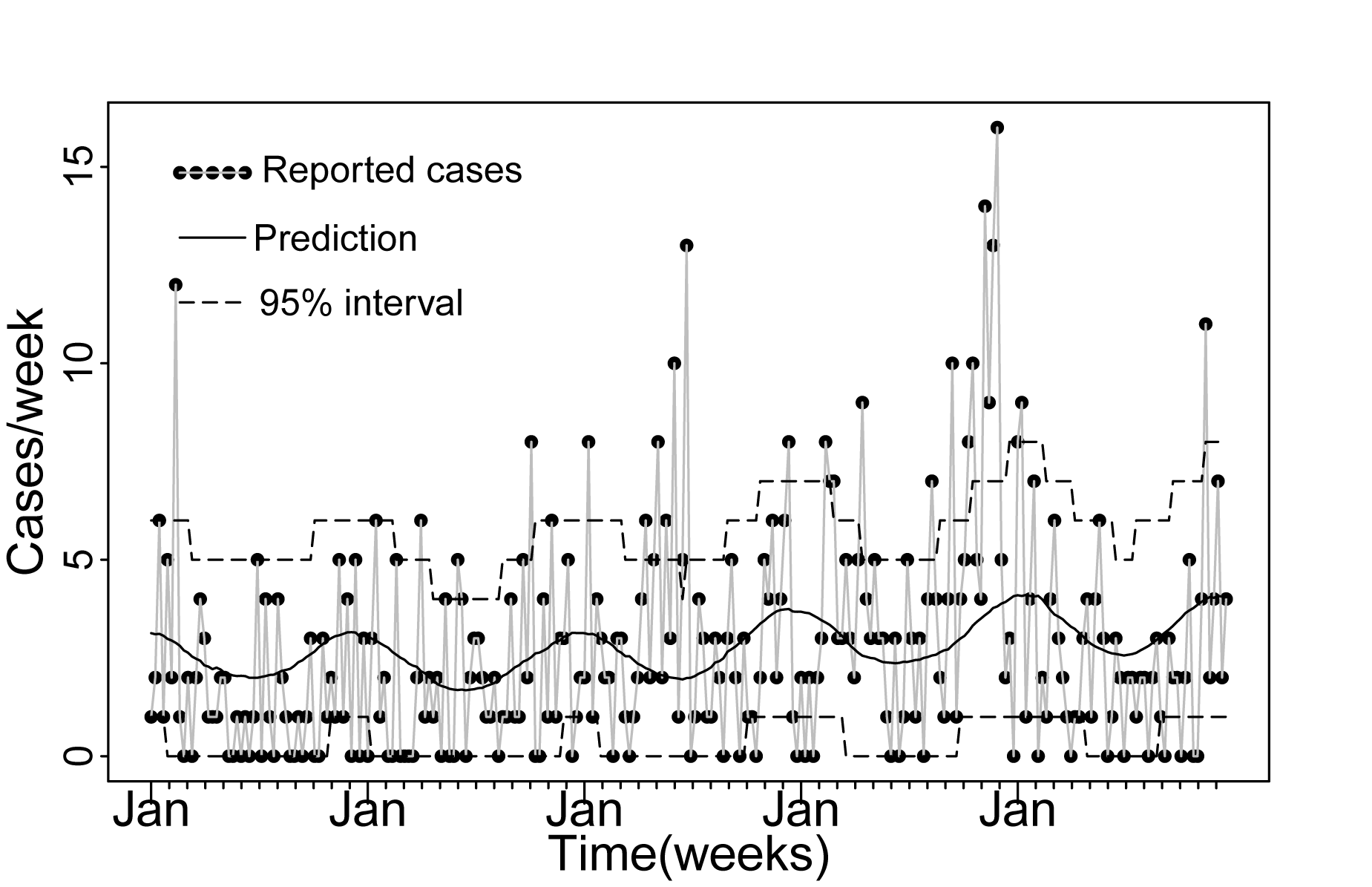

Supplement: Figure S3 — Fitted baseline and one sided 95% confidence interval for weekly counts for case RespSy Years 2006–2010. Poisson regression using model: counts ∼ sin(2π t) + cos(2π t) + log(histmean). (TIF) [file pone.0111335.s003.tif]

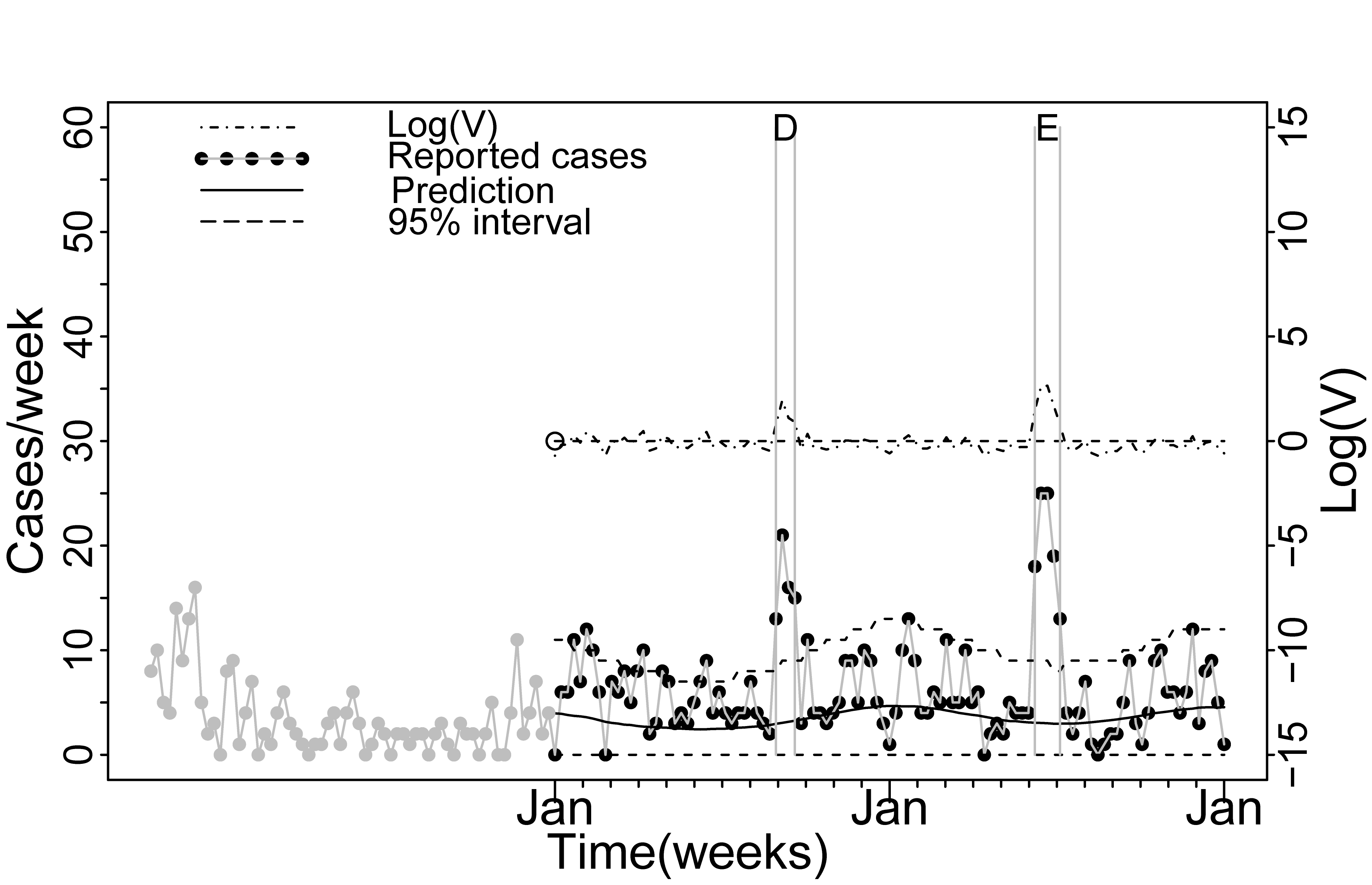

Supplement: Figure S4 — Application of RespSy NB-model on the fictive test dataset. The vertical lines bounds peaks inserted during Year 1, week 36 to 39 (D), Year 2, week 24 to 28 (E). The gray points indicate historical data used to calculate the historical average (histmean). (TIF) [file pone.0111335.s004.tif]

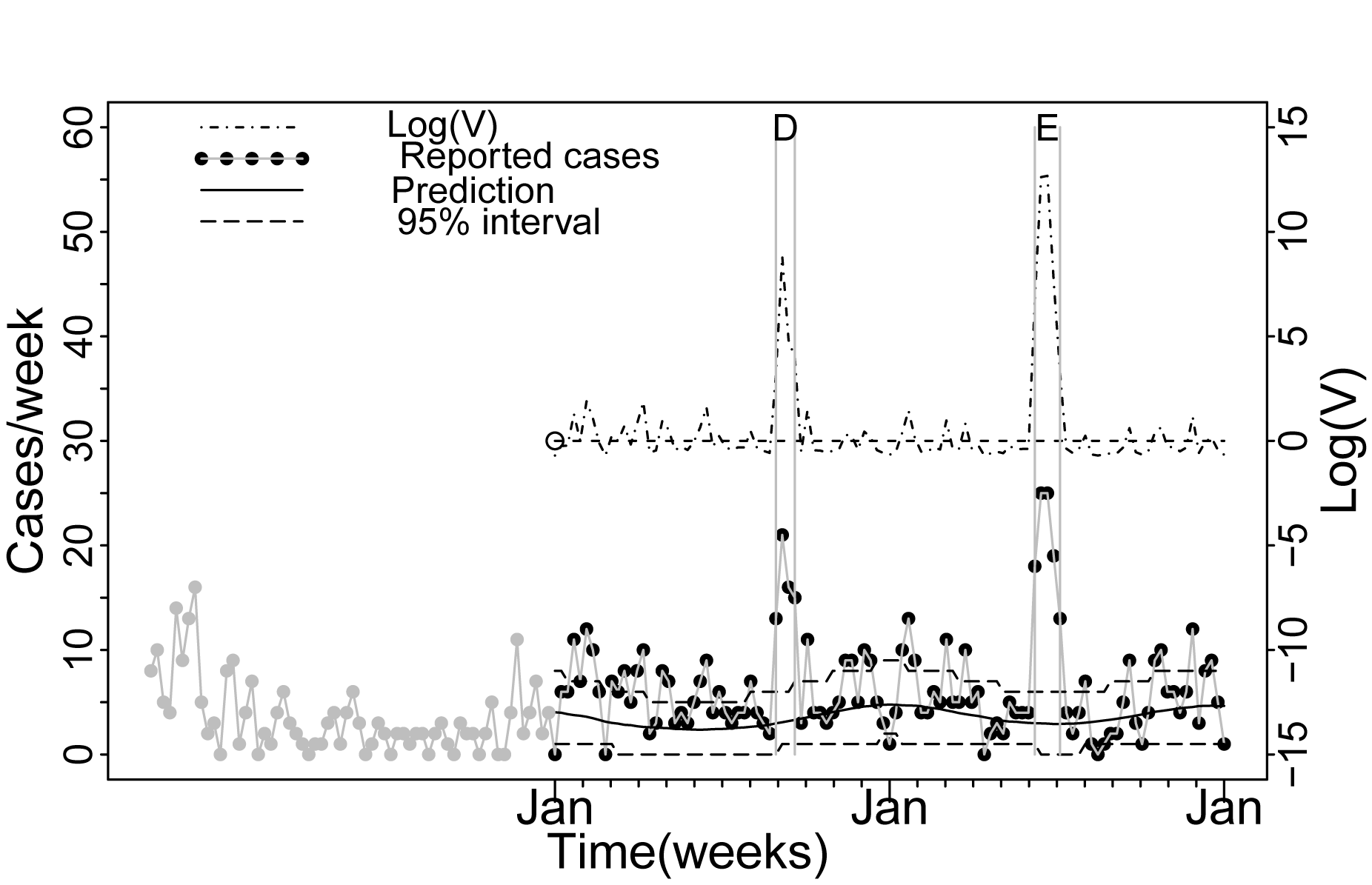

Supplement: Figure S5 — Application of RespSy Poisson-model on the fictive test dataset. The vertical lines bounds peaks inserted during Year 1, week 36 to 39 (D), Year 2, week 24 to 28 (E). The gray points indicate historical data used to calculate the historical average (histmean). (TIF) [file pone.0111335.s005.tif]
